# Data driven fuel consumption prediction model for green aviation using radial basis function neural network

**DOI:** 10.1038/s41598-025-11941-8

**Published:** 2025-07-19

**Authors:** Yuandi Zhao, Zhongyi Wang, Xiaohui Wang, Ye Song, Yuzhe Han

**Affiliations:** 1 College of Air Traffic Management, Civil Aviation University of China, Tianjin, 300300 China; 2 College of Computer Science and Technology, Civil Aviation University of China, Tianjin, 300300 China

**Keywords:** Green civil aviation, Fuel consumption, Radial basis function neural network, Fuel penalty for carrying additional fuel, Aerospace engineering, Energy conservation

## Abstract

In response to the growing demand for sustainable aviation, a fuel consumption prediction model based on Radial Basis Function (RBF) Neural Networks was proposed. Using high-resolution onboard Quick Access Recorder (QAR) data, which contains richer flight parameters and higher accuracy, RBF models were constructed based on the extracted key influencing factors for different flight phases, including takeoff/climb, cruise, and descent/approach. The model provides a lightweight and computationally efficient solution for high-dimensional, nonlinear flight data, ensuring accuracy with lower computational burdens. It is suitable both for pre-flight ground-based fuel consumption prediction and deployment in resource-constrained onboard environments, enabling real-time prediction during flight operations. Experimental results showed that the RBF model’s prediction errors for the takeoff/climb, cruise, and descent/approach phases were 5.73%, 3.36%, and 14.04%, respectively, significantly outperforming the comparison models. The error variances from ten-fold cross-validation were 0.31%, 0.15%, and 0.29%, respectively, confirming the robustness of the model. Further analysis indicated that the model can be employed to evaluate the “fuel penalty for carrying additional fuel” patterns and enhance fuel efficiency. This study provided valuable insights and theoretical support for airlines in optimizing flight planning and minimizing fuel consumption, thereby contributing to the sustainable development of green aviation.

## Introduction

With the ongoing economic development and the rapid growth of the aviation industry, greenhouse gas emissions have become a significant factor in global climate change. At the 41st Assembly of the International Civil Aviation Organization (ICAO), a long-term goal was adopted, aiming for net-zero carbon emissions in the international aviation sector by 2050^1^. Additionally, energy costs, particularly fuel, account for a significant proportion of airline operating costs, around 30% of their core business expenses. As a result, airlines have an inherent motivation to engage in fuel-saving efforts. Therefore, developing more accurate aircraft fuel consumption prediction models, improving fuel efficiency, and reducing carbon emissions have become critical research issues for airlines^2^. However, accurately predicting aircraft fuel consumption remains challenging due to the complex interplay of various factors, such as aircraft performance, flight operations, meteorological conditions, and air traffic flow.

A significant amount of research on aircraft fuel consumption has been conducted, which can be broadly classified into three categories: studies based on aircraft performance, studies using statistical methods, and studies utilizing machine learning techniques. The representative studies for each category are listed in Table 1.

Research based on aircraft performance primarily focuses on analyzing the inherent relationships between fuel consumption and design parameters such as the aircraft’s propulsion system, aerodynamic performance, and structural weight. Zhao Yifei et al. defined a terminal area arrival flight efficiency index based on fuel consumption, and used the Base of Aircraft Data (BADA) fuel consumption model to calculate the fuel consumption of arrival flights^3^. Song Hanbing et al. developed a fuel consumption and pollutant emission estimation model for various flight phases based on the ICAO’s standard takeoff and landing cycle model and Boeing Method 2 model^4^. Yuan Lyu et al. proposed a hybrid data-driven model that maps flight operation data to aircraft performance analysis in order to predict fuel consumption and flight status information^5^. These methods, while theoretically capable of deriving fuel consumption patterns related to aircraft design, are often limited to the specific design and flight modes of the aircraft.

Studies using statistical methods to analyze aircraft fuel consumption primarily rely on historical data and employ techniques such as regression analysis and analysis of variance to identify and quantify the key factors influencing fuel consumption. Lei Kang et al. developed a fuel estimation method based on an econometric model, which derives gate fuel data from pushback weight and fuel consumption and proposes a corresponding predictability metric^6^. Qian Yu et al. utilized Quick Access Recorder (QAR) data from an airline, applying linear regression methods to fit taxi-out time and establish a fuel consumption model for the taxi phase^7^. Thowayeb H. Hassan et al. used generalized linear models to assess total fuel consumption predictions^8^. These methods are capable of uncovering patterns from actual data but often overlook the nonlinear relationships of systemic factors.

With the advancement of computational power, machine learning techniques have gradually become an important tool for researching aircraft fuel consumption. Gu Runping et al. employed an improved Back Propagation (BP) Neural Network to establish a landing residual fuel prediction model and introduced a genetic algorithm to optimize the network’s initial weights and thresholds, achieving higher prediction accuracy and enhancing the monitoring of fuel levels at landing^9^. Chen Jingjie et al. proposed an estimation method for aircraft fuel consumption intervals based on data deviation and density distribution under sampling, and used a relevance vector machine to establish the fuel consumption interval estimation model^10^. Huang Chenyu et al. utilized Classification and Regression Trees (CART) and Neural Networks to establish a fuel consumption estimation model, which was validated using data from flight data recorders (FDR) and Automatic Dependent Surveillance-Broadcast (ADS-B) systems. This approach enabled more efficient and cost-effective fuel consumption estimation, thereby improving fuel management and monitoring in airline operations^11^. Wang Ziming et al. utilized QAR data and applied a random forest model to predict aircraft fuel consumption rates. They developed dedicated prediction models tailored to different aircraft types and flight phases, thereby enhancing the accuracy of fuel estimation^12^. Feng Yuxuan et al. proposed a deep learning model called FCPNet, based on multimodal data that integrates route, weather, and operational information. The model employs Graph Convolutional Networks (GCN), Convolutional Long Short-Term Memory (ConvLSTM) architectures for feature extraction, and incorporates attention mechanism along with binary encoding methods to achieve pre-flight fuel consumption prediction^13^. Tang Weizheng et al. optimized the hyperparameters of the Bidirectional Long-Short-Term Memory network–Kolmogorov-Arnold network (BiLSTM–KAN) model using an Improved Arctic Puffin Optimization (IAPO). They combined the prediction results of individual sub-sequences to obtain the final fuel consumption forecast^14^. In addition, machine learning techniques have made significant strides in fuel consumption prediction in other transportation domains, such as the automotive industry. Sasanka Katreddi et al. modeled the fuel consumption of modern heavy-duty trucks using Artificial Neural Network (ANN), predicting total trip fuel consumption and instantaneous fuel consumption with a limited set of key parameters^15^. Sasanka Katreddi et al. further provided a comprehensive review of the various applications of artificial intelligence in heavy-duty truck operations^16^. Rafael Canal et al., utilized real-world Electronic Control Unit (ECU) data, and applied three machine learning algorithms to classify driver behavior in terms of fuel efficiency. They also compared the performance of different algorithms in fuel consumption prediction tasks^17^.

In the field of aircraft fuel consumption efficiency evaluation, extensive research has been conducted. Wang Lei, based on radar trajectory data, proposed a continuous altitude difference change algorithm for flight state identification, and constructed a multi-parameter aircraft fuel consumption model using an echo state network. This model was further applied to analyze air traffic operational fuel efficiency based on radar trajectories^18^. Hu Rong and colleagues, based on the BADA aircraft performance model and considering meteorological factors, established a fuel consumption correction model for the aircraft’s approach phase. They discussed the effects of temperature, pressure, and wind speed variations on aircraft fuel efficiency from the perspectives of fuel flow and fuel consumption^19^. Huang Ganxiang and others used the fuel consumption intensity index measured in ton-kilometers to analyze the fuel efficiency of China’s air transport industry^20^.

**Table 1:** Fuel consumption prediction methods.

Methods	Advantages		Disadvantages	References
Aircraft performance	1) Aircraft performance parameter-based approach; 2) Simple model structure; 3) Easy implementation		1) Limited and simple data sources; 2) Theoretical-only fuel estimates; 3) Applicability restricted to ideal conditions	^3^Zhao et al.,2020; ^4^Song et al.,2020; ^5^Lyu et al.,2020
Statistical methods	1) Real data-based approach; 2) Low computational cost		1) Lack of flight phase segmentation; 2) Inability to capture nonlinear relationships across different flight phases	^6^Lei et al., 2020; ^7^Qian et al., 2018; ^8^Hassan et al.,2020
Machine learning	1) Ability to handle high-dimensional and nonlinear data; 2) High prediction accuracy		1) Requirement for large datasets and high computational resources; 2) Unsuitability for onboard predictions	^9^Gu et al., 2020; ^10^Chen et al., 2021; ^11^Huang et al., 2022; ^12^Wang et al., 2025; ^13^Lin et al., 2024; ^14^Tang et al., 2025

The above analysis indicates that existing research methods still exhibit varying degrees of limitations in terms of data quality, flight phase modeling strategies, and model efficiency. These limitations hinder their ability to fully meet the comprehensive requirements for prediction accuracy and real-time performance in actual flight operations. To address these issues, this study introduces the following three advancements:

1. Data Perspective: High-resolution onboard QAR data is utilized, which offers richer flight parameters and higher accuracy. This significantly enhances the authenticity and completeness of model inputs, effectively overcoming the limited dimensionality and accuracy shortcomings of traditional aircraft performance data and ADS-B data.
2. Modeling Strategy: A phase-based modeling approach is adopted to reflect the distinct fuel consumption characteristics of different flight segments, including takeoff/climb, cruise, and descent/approach. This allows for a more precise representation of fuel-influencing mechanisms across phases, thereby improving the model’s prediction accuracy and generalization capability.
3. Model Selection and Application: A lightweight and computationally efficient Radial Basis Function (RBF) Neural Network was employed. This model effectively handles high-dimensional, nonlinear flight data, ensuring high prediction accuracy while providing strong real-time performance and practical feasibility of deployment. It is suitable both for ground-based fuel consumption prediction prior to flights and for deployment in resource-constrained onboard environments, enabling real-time prediction during flight operations.

Furthermore, this paper explores the “fuel consumption due to extra fuel” patterns in depth, analyzing the intrinsic relationship between “excess fuel” and “excessive fuel consumption”. This provides a theoretical basis for airlines to reduce fuel consumption and optimize flight planning, thereby contributing to lower operational costs and enhancing the sustainability of green aviation.

## Methodology

### Extraction and analysis of influencing factors

QAR data, recording high-resolution and comprehensive flight parameters with high accuracy, were selected for this study to provide a reliable and rich data source for fuel consumption modeling. Relevant influencing factors and the fuel flow of the left and right engines of the aircraft were extracted from the QAR data of a specific airline’s flight from Beijing Daxing International Airport (PKX) to Guangzhou Baiyun International Airport (CAN). The extracted factors include longitude, latitude, standard pressure altitude, ground speed, true heading, Mach number, vertical acceleration, lateral acceleration, longitudinal acceleration, and other 15 factors that affect aircraft fuel consumption. These factors which extracted from the QAR, along with the fuel flow of the left and right engines, are presented in Table 2.

**Table 2:** Description table of extracted parameter.

Serial number	Parameter	Description	Unit of measurement
1	GS	Ground speed	knot
2	MACH	Mach number	mach
3	VRTG	Vertical acceleration	g
4	LATG	Lateral acceleration	g
5	LONG	Longitudinal acceleration	g
6	LONPC	Present position longitude	deg
7	LATPC	Present position latitude	deg
8	SA	Standard pressure altitude	feet
9	TH	True heading	deg
10	TAT	Total air temperature	deg C
11	WS	Wind speed	knot
12	TWD	True wind direction	deg
13	EGT	Exhaust gas temperature	deg C
14	GW	Gross weight	1b
15	TFQ	Total fuel quantity	t
16	FF1	Fuel flow engine 1	b/h
17	FF2	Fuel flow engine 2	b/h
18	FF	Fuel flow	b/h

**Fig. 1:**
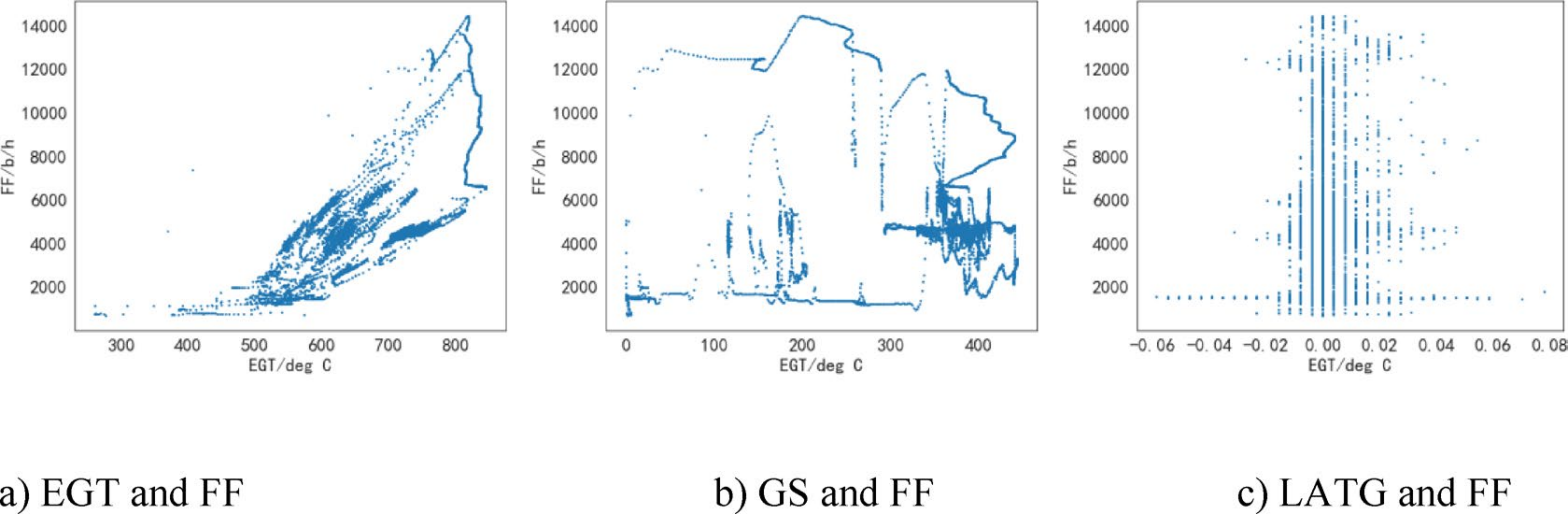
Relationships between different influencing factors and FF.

The EGT data, GS data and LATG data extracted from the QAR will be compared and analyzed against FF as shown in Fig. 1.

The comparison and analysis of EGT data with the FF are depicted in Fig. 1a. The horizontal axis represents the EGT, and the vertical axis represents the FF. It can be observed that as the EGT increases, there is a noticeable increase in the FF, indicating a clear positive correlation between EGT and FF. Additionally, factors such as LONPC, LATPC, LONG, GW, and TFQ exhibit similar relationships with the FF.

The comparison and analysis of aircraft GS data with the FF are depicted in Fig. 1b. The horizontal axis represents the GS of the aircraft, while the vertical axis represents the FF. It can be observed that there is no significant trend in the FF with increasing GS. This indicates a weak linear relationship between GS and FF, suggesting a lower degree of fit. Further analysis is required to better understand this relationship. Similarly, factors such as MACH, WS, SA, TH, and TAT exhibit similar relationships with the FF.

The comparison and analysis of aircraft LATG data with the FF are illustrated in Fig. 1c. It can be observed that the LATG values are mostly concentrated between − 0.1 and 0.1. This is due to the limitation of the data in this study, as the flights considered in the study follow a single flight path. As a result, it does not adequately reflect the relationship between LATG and FF. Therefore, further consideration of LATG is not warranted. Similarly, factors such as VRTG and TWD exhibit similar relationships with the FF.

Due to the limitation and concentration of the values of LATG, VRTG, and TWD, these factors will not be further considered in this study. However, the factors of EGT, LONPC, LATPC, LONG, GW, TFQ, GS, Mach, WS, SA, TH, and TAT show correlation with the FF and their specific relationships need to be further analyzed.

### Analysis of key factors across flight phases

Given the significant variations in flight attitude and operational conditions across different flight phases, which are primarily influenced by weather conditions, aircraft performance, and other relevant factors, the determinants of fuel consumption also vary accordingly. Therefore, this study divides the flight process into takeoff/climb, cruise, and descent/approach phases and conducts a detailed analysis of the influencing factors in each phase.

Principal Component Analysis (PCA) is a multivariate statistical method that uses dimensionality reduction techniques to transform multiple variables into a few principal components^21^. Its advantage lies in eliminating the correlation between factors, but the drawback is that the computation process loses some of the variables’ original meanings. Grey Relational Analysis (GRA) is a method to assess the correlation degree between factors based on the similarity of the geometric shapes of their change curves^22^. However, it is subjective in nature. Therefore, a combination of these two factor analysis methods is used to screen the key factors that affect aircraft fuel consumption.

**Fig. 2:**
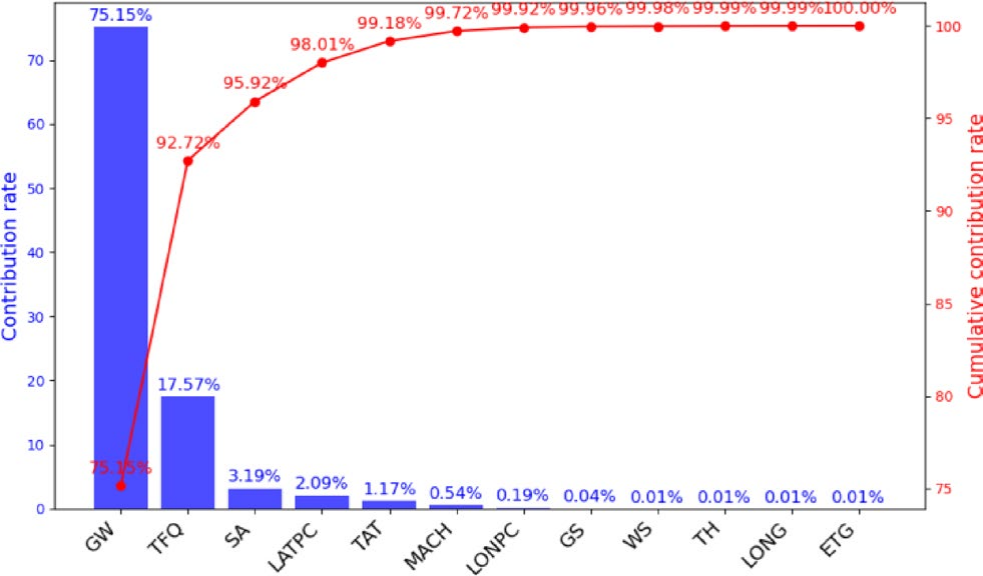
Principal component contribution.

PCA is applied to reduce the dimensionality of the 12 fuel consumption influencing factors. By analyzing the loadings of the original variables on the principal components, the contribution of each factor is determined and ranked, as presented in Fig. 2. The PCA results of the three flight phases are consistent, showing that the cumulative contribution rate of the top 10 factors reaches 99.99%, indicating that they retain almost all the information from the original dataset.

**Fig. 3:**
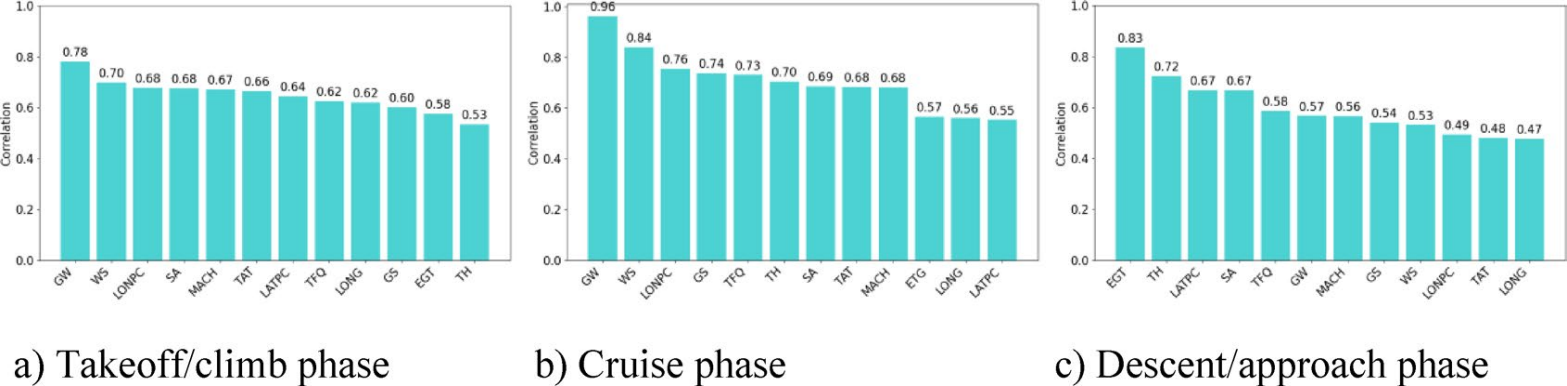
Grey relational.

GRA is employed to analyze the 12 fuel consumption influencing factors in the takeoff/climb phase. The results, ranked according to their grey relational degrees, are presented in Fig. 3. During the takeoff/climb phase, most factors have grey relational scores between 0.6 and 0.7, with a few factors falling below 0.6. In the cruise phase, most factors score above 0.6, although a few remain below 0.6. In the descent/approach phase, the majority of factors exhibit grey relational scores above 0.5, with a few factors scoring below 0.5.

Each method has its own advantages and limitations. By integrating the results of both analyses, the key factors influencing fuel consumption can be effectively identified. The comprehensive correlation is defined as follows, according to Equation (1):

1 $$\alpha = \mu \left| \beta \right| + \left( {1 - \mu } \right)\left| \gamma \right|$$

where *α* represents the comprehensive correlation, *β* represents the principal component score, *γ* represents the grey correlation coefficient, and *µ* represents the weights assigned to each component.

**Fig. 4:**
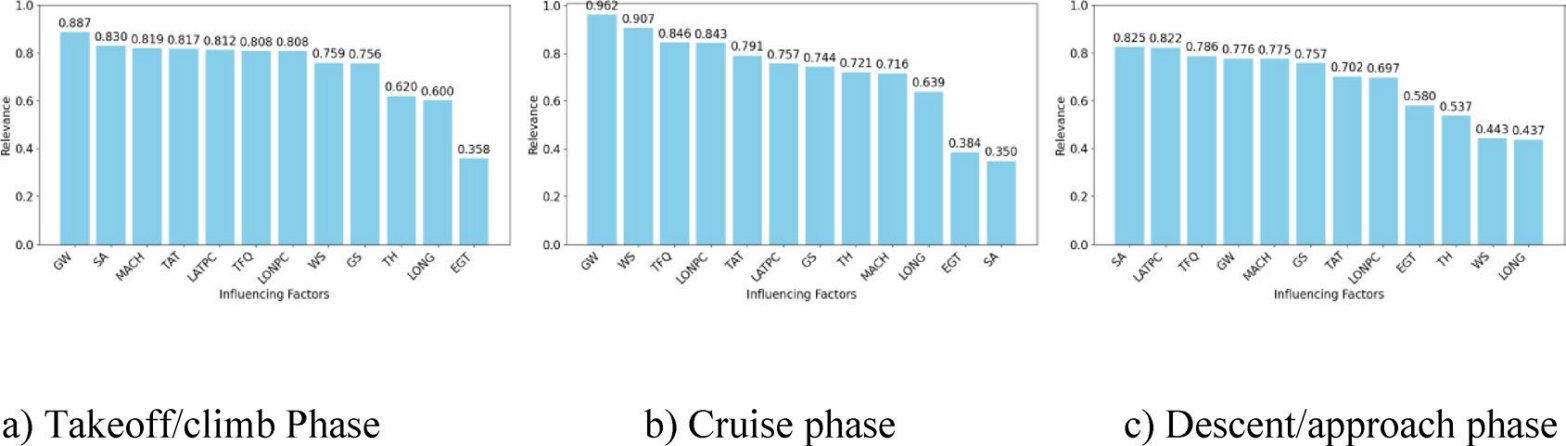
Comprehensive correlation.

Due to the different perspectives of the two methods, µ is set to 0.5 in this case. The comprehensive correlation of each factor is calculated according to Eq. (1), and the results are shown in Fig. 4.

Based on the analysis conducted, the key factors affecting fuel consumption during the tactical phase are summarized in Table 3.

**Table 3:** Key influencing factors at each phase.

Flight phase	Influencing factors
Takeoff/climb phase	GW, SA, MACH, TAT, LATPC, TFQ, LONPC, WS, and GS
Cruise phase	GW, WS, TFQ, LONPC, TAT, LATPC, GS, TH, and MACH
Descent/approach phase	SA, LATPC, TFQ, GW, MACH, GS, MACH, GS, TAT, LONPC, and EGT

### Fuel consumption prediction model based on RBF neural network

Traditional fuel consumption prediction methods struggle to capture the nonlinear and complex characteristics of flight data. Although deep learning models possess strong modeling capabilities, their training processes require multi-layer weight updates via backpropagation and rely heavily on large-scale data and computational resources, which poses challenges for practical deployment. In contrast, In contrast, RBF Neural Network is lightweight and computationally efficient, and it effectively models high-dimensional, nonlinear data. By applying nonlinear kernel functions, it maps the original input into a transformed feature space, and solves the output weights through matrix operations. This approach maintains strong modeling capabilities while significantly reducing computational costs, making it more suitable for deployment in resource-constrained flight environments. Therefore, this study employs an RBF Neural Network^23^ to model aircraft fuel consumption.

**Fig. 5:**
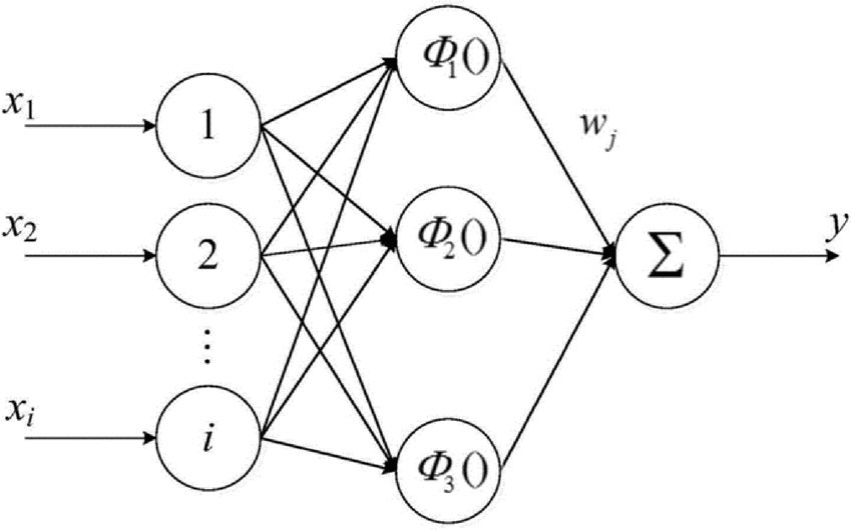
Structure diagram of RBF Neural Network.

The RBF Neural Network is designed as a three-layer network comprising an input layer, a hidden layer, and an output layer, as depicted in Fig. 5. The key influencing factors are entered into the network through the input layer, processed by the hidden layer, and the predicted values are obtained through the output layer.

The relationship between the input and output of the RBF Neural Network model is represented by Eq. (2):

2 $$\:y_{i} = \sum {\:_{{i = 1}}^{n} } w_{j} \times \:\exp \left( {\frac{1}{{ - 2\delta \:^{2} }}\left\| {x_{p} - x_{i} } \right\|^{2} } \right)$$

where $\:{y}_{i}$represents the output of the model, $\:{x}_{i}$ represents the input of the model, $\:{x}_{p}$ represents the center of the radial basis function, $\:{w}_{j}\:$represents the weight value, and $\:\delta\:$ represents the smoothing parameter.

## Case study

Key factors affecting fuel consumption during different flight phases, as identified from QAR data, are chosen as inputs for the model. The dataset comprises 44 flights, each containing approximately 12,000 trajectory points.

The RBF Neural Network is then employed for fuel consumption prediction and analysis. The parameters involved in the experiment include the radial basis function width (*δ*) and the number of hidden layer neurons (*n*).

### Influence of radial basis function width

In an RBF network, the selection of *δ* is a critical factor influencing the prediction performance. A larger *δ* results in a smoother fitting curve for the model, reducing its complexity. However, this can lead to underfitting, where the model fails to capture detailed features in the data. Conversely, a smaller *δ* makes the model more complex, enabling it to fit the training data more precisely, but it may lead to overfitting. By fixing the number of hidden layer neurons and selecting different values for *δ*, the error rate of the prediction is computed, as shown in Table 4. The results indicate that when *δ* is set to 0.34, the prediction error rate is minimized across all flight phases. The experiment demonstrates that an appropriately chosen *δ* can achieve a good balance between fitting ability and generalization capability, thereby reducing prediction errors and enhancing prediction accuracy. Consequently, in this study, a radial basis function width of 0.34 is selected for the prediction analysis.

**Table 4:** Comparison of error rates for different *δ*.

δ	Takeoff/climb phase	Cruise phase	Descent/approach phase
0.30	7.20%	4.03%	15.85%
0.32	7.04%	3.86%	14.94%
**0.34**	**5.73%**	**3.36%**	**14.04%**
0.36	6.99%	3.52%	15.02%
0.38	7.00%	3.69%	15.22%

### Influence of the number of hidden layer neurons

The number of *n* has a significant impact on the model’s fitting performance. Increasing the number of neurons typically enhances the model’s ability to capture more data features, improving its fitting capacity. However, an excessive number of neurons may lead to an overly complex model, causing overfitting. In contrast, fewer neurons help prevent overfitting but may result in an insufficiently complex model, leading to underfitting. By fixing the radial basis function width and selecting different *n*, various prediction results are obtained, as shown in Table 5. The experimental results demonstrate that an appropriately chosen number of hidden neurons strikes a balance between the model’s fitting ability and generalization capacity, leading to better prediction performance. It can be observed from Table 5 that when *n* is set to 4000 for the takeoff/climb and cruise phases, and *n* is set to 2000 for the descent/approach phase, the prediction error rate reaches its minimum. Therefore, for the takeoff/climb and cruise phases, 4000 hidden layer neurons are selected, while for the descent/approach phase, 2000 hidden layer neurons are chosen for the prediction analysis.

**Table 5:** Comparison of prediction results with different *n*.

*n*	Takeoff/climb phase	Cruise phase	*n*	Descent/approach phase
3200	8.17%	4.37%	1200	16.09%
3600	6.17%	3.65%	1600	14.34%
**4000**	**5.73%**	**3.36%**	**2000**	**14.04%**
4400	5.81%	3.72%	2400	16.15%
4800	6.52%	4.12%	2800	16.65%

### Comparative analysis

**Fig. 6:**
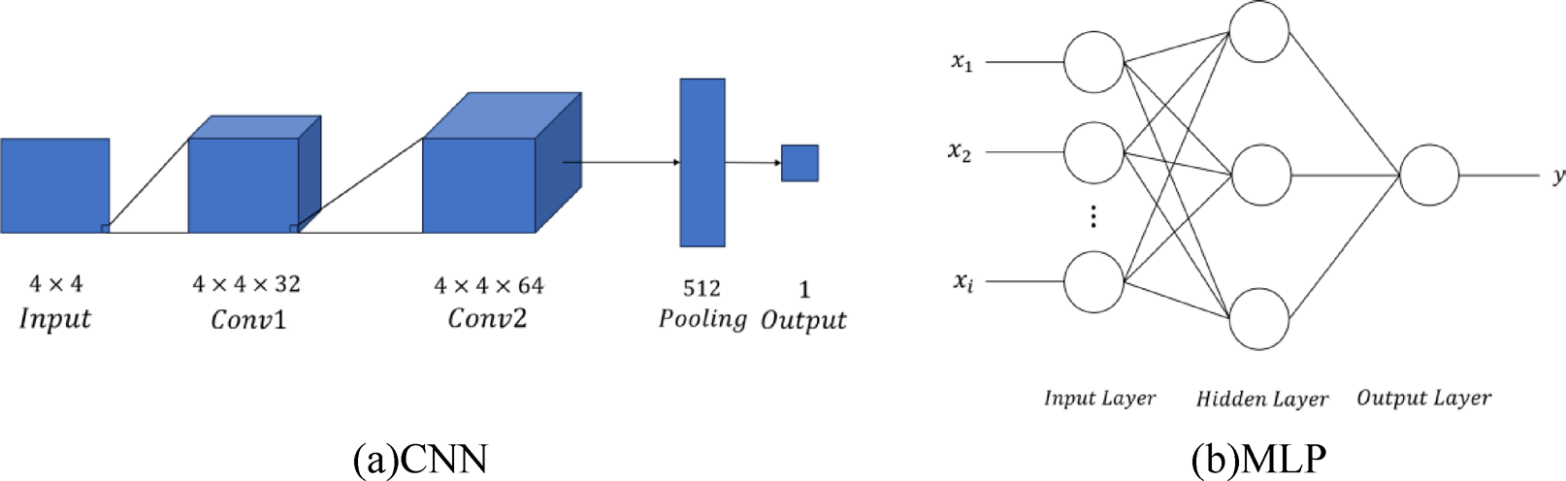
Structure of CNN and MLP.

**Fig. 7:**
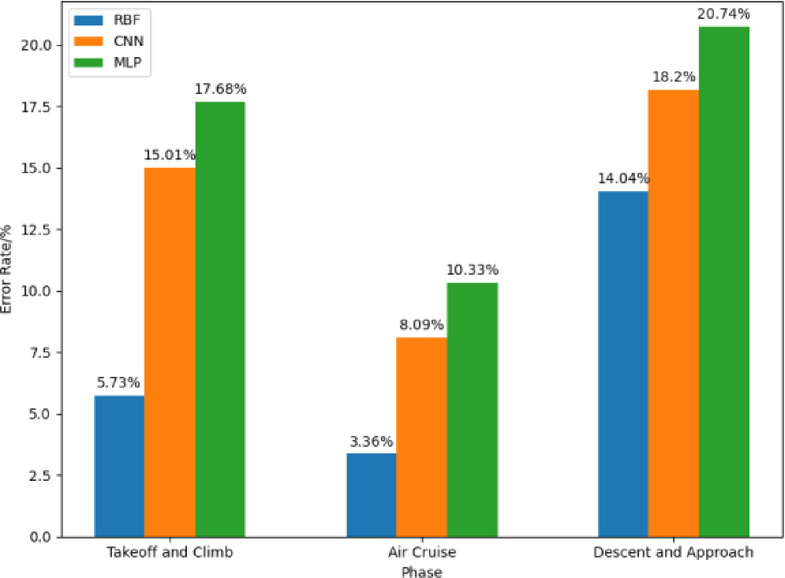
Comparison of error rate.

The key factors influencing fuel consumption in different flight phases from the QAR data are separately input into the RBF Neural Network fuel consumption prediction model. The predicted results are obtained, and compared with the results from the Convolutional Neural Network (CNN) models^24^and Multi-Layer Perceptron (MLP) models^25^. All three models were trained and tested on the same dataset to ensure consistency and fairness in comparison. The CNN model consists of two convolutional layers, one pooling layer, and an output layer, as shown in Fig. 6a. The MLP model comprises a single hidden layer with three hidden neurons, as illustrated in Fig. 6(b). The prediction error rates are shown in Fig. 7.

From Fig. 7, it can be observed that in the takeoff and climb phase, the average error rate of the RBF Neural Network model is 5.73%, which is lower than the CNN model’s 15.01% and the MLP model’s 17.68%.

**Fig. 8:**
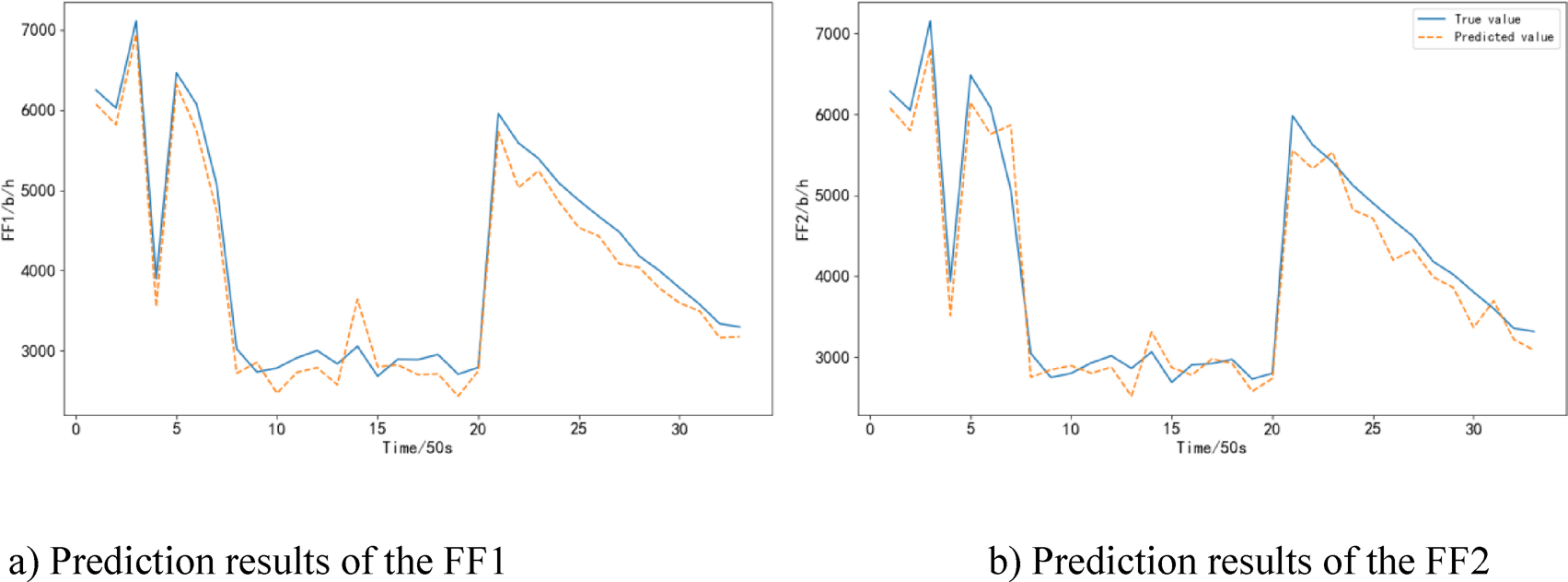
Prediction results of the FF in take-off and climb phase.

The predicted results are shown in Fig. 8. It can be seen that the RBF Neural Network model can effectively predict the fuel consumption of the aircraft during the climb phase, and it is relatively close to the actual values. However, there are occasional fluctuations in the predictions.

During the cruise phase, the RBF Neural Network model has an average error rate of 3.36%, which is lower than the CNN model’s 8.09% and the MLP model’s 10.33%.

**Fig. 9:**
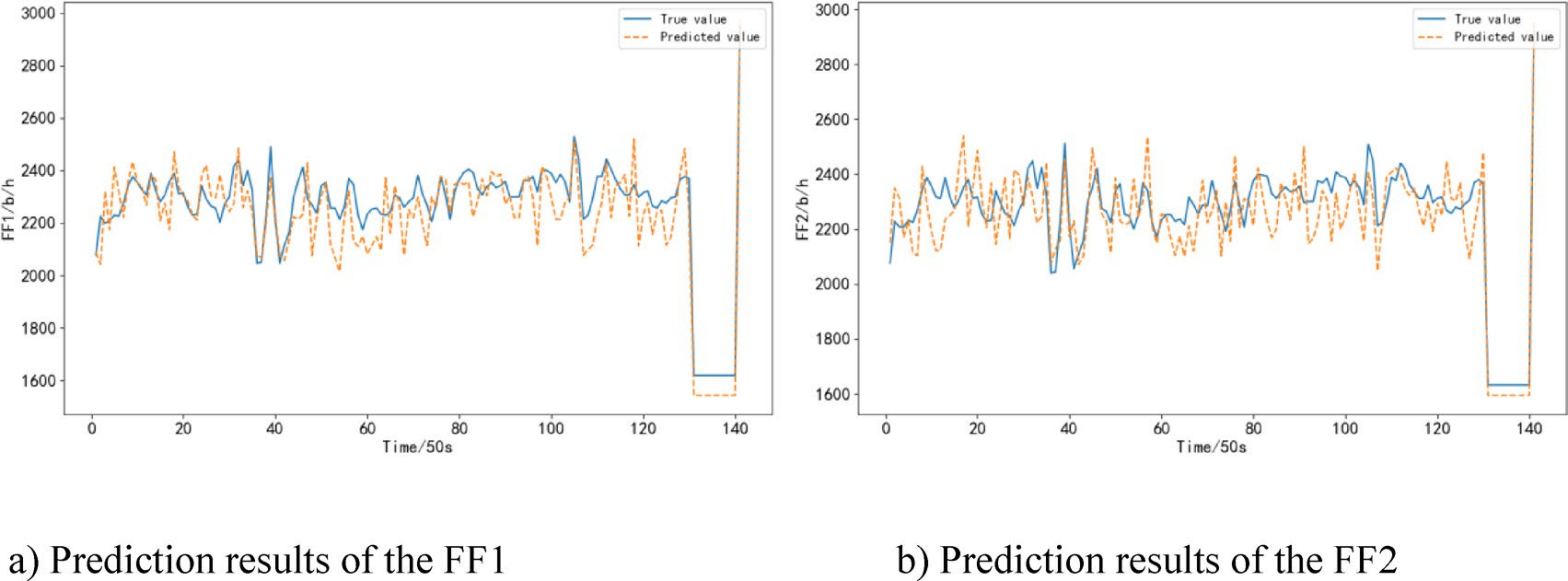
Prediction results of the FF in cruise phase.

The predicted results are shown in Fig. 9. It can be seen that the RBF Neural Network model more accurately predicts the fuel consumption of the aircraft during the cruise phase. It is worth noting that fuel consumption, as a variable influenced by multiple flight conditions and external environmental factors, inherently exhibits significant volatility. This volatility can also be reflected in the model’s prediction results, potentially leading to a certain degree of instability in the predicted fuel consumption curves.

During the descent/approach phase, the RBF Neural Network model has an average error rate of 14.04%, which is lower than the CNN model’s 18.20% and the MLP model’s 20.74%.

**Fig. 10:**
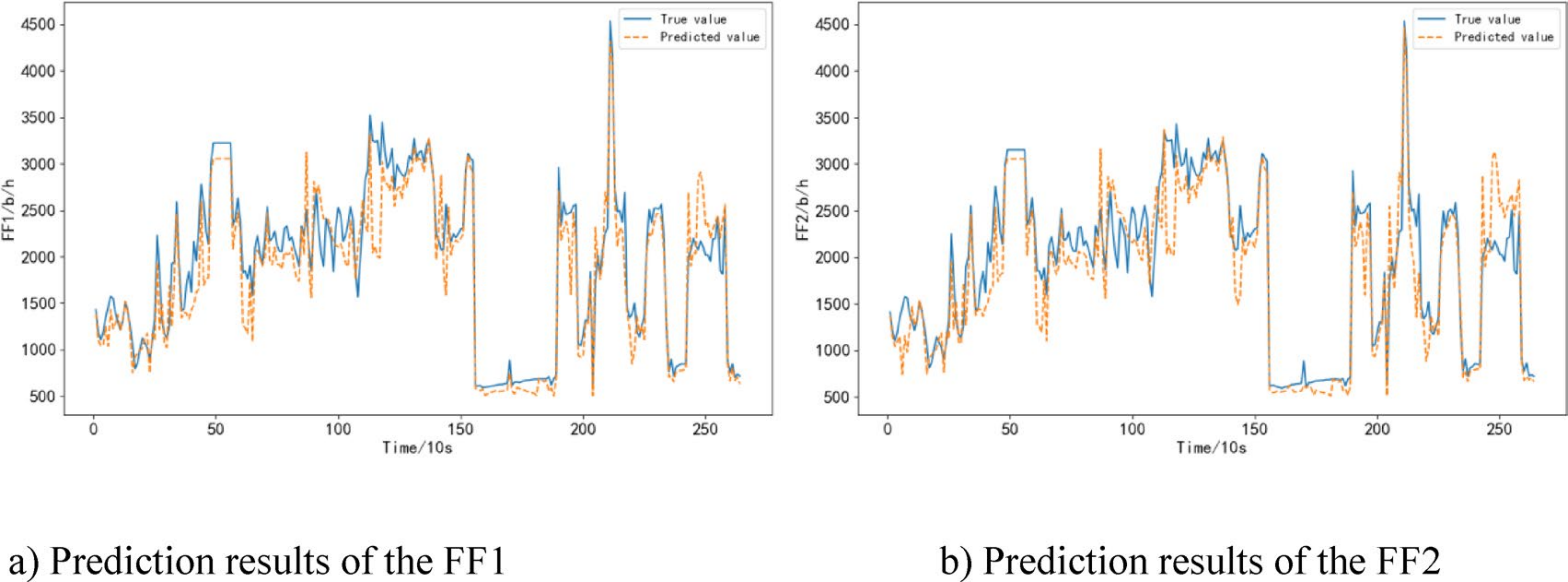
Prediction results of the FF in the descent/approach phase.

The predicted results are shown in Fig. 10. It can be observed that the RBF Neural Network model exhibits larger prediction errors during the descent/approach phase compared to the cruise phase. However, it still outperforms the other models in terms of accuracy.

This is because during the cruise phase, the aircraft is in a relatively stable flight state with fewer maneuvering actions. Moreover, the cruise phase constitutes a significant proportion of the entire flight duration. Therefore, the overall prediction error rate is lower during the cruise phase. Among the models, the RBF Neural Network model exhibits the smallest prediction error.

On the other hand, during the descent/approach phase, the aircraft is under the control of air traffic controllers, leading to more maneuvering actions and frequent changes in flight conditions. Additionally, this phase is relatively short in duration. As a result, the descent/approach phase generally exhibits higher prediction error rates. However, even in this phase, the RBF Neural Network model has the smallest prediction error among the three models, indicating that the RBF Neural Network model outperforms the other two models in terms of predictive performance.

In summary, the comparison with CNN and MLP models reveals that the RBF Neural Network consistently achieves lower prediction errors across all three flight phases, with its advantages particularly pronounced during the takeoff/climb and cruise phases. This indicates that the RBF model has stronger modeling and generalization capabilities when handling flight phases characterized by significant nonlinear features and relatively stable states. Although the prediction error in the descent/approach phase is relatively higher, the RBF model still outperforms the other models, reflecting its adaptability to complex and dynamic flight conditions. Overall, the results validate the effectiveness and superiority of the RBF network in aircraft fuel consumption prediction.

### Analysis of influencing factor set validation

Based on the availability of data, the influencing factors have been divided into three parameter sets: Few Parameters Set, Moderate Parameters Set, and Many Parameters Set. The Five influencing factors in the Few Parameters Set can be obtained from ADS-B data, making it an easily accessible dataset. ADS-B is an automated broadcasting technology for use in aviation that provides real-time precise position information and environmental awareness. The Eight influencing factors in the Moderate Parameters Set can be obtained from both ADS-B data and meteorological data, making it a relatively easy dataset to obtain. The Many Parameters Set includes some influencing factors that can only be obtained from QAR data, making it a less accessible dataset.

Based on the types of parameters and the difficulty in obtaining them, the set of influencing factors has been redefined, as shown in Table 6.

**Table 6:** Parameter set table.

	Parameter composition
Few parameters set	GS, LONPC, LATPC, SA, and TH
Moderate parameters set	GS, LONPC, LATPC, SA, TH, WS, TWD, and TAT
Many parameters set	GS, LONPC, LATPC, SA, TH, WS, TWD, TAT, MACH, VRTG, LATG, LONG, EGT, GW, and TFQ

These three parameter sets are separately input into the RBF Neural Network fuel consumption prediction models for each flight phase. The predicted results are compared with those obtained from CNN and MLP models, and the prediction errors are shown in Table 7.

**Table 7:** Comparison of error rates for different parameter sets.

	takeoff/climb phase			cruise phase			descent/approach phase		
	RBF	CNN	MLP	RBF	CNN	MLP	RBF	CNN	MLP
Few Parameters Set	5.58%	19.18%	16.41%	8.55%	19.42%	12.37%	27.16%	44.21%	40.46%
Moderate Parameters Set	4.63%	8.87%	13.97%	2.54%	9.07%	8.31%	25.57%	51.77%	38.49%
**Many Parameters Set**	**1.70%**	3.15%	3.47%	**0.49%**	3.36%	3.43%	**9.17%**	18.38%	9.42%

Significant values are in bold.

From Table 7, it can be observed that when using the set of few parameters as input, all three models exhibit relatively large prediction error rates. As the number of parameters increases, the prediction errors for all models gradually decrease. Among them, the RBF Neural Network fuel consumption prediction model shows the smallest prediction error, indicating superior prediction performance compared to the other two models.

In terms of flight phases, the descent/approach phase involves more maneuvering actions and frequent changes in flight status, which results in relatively higher overall prediction error rates. On the other hand, the cruise phase exhibits more stable flight conditions with fewer maneuvering actions, and it also constitutes a larger proportion of the overall flight duration. Therefore, the prediction error rates for the cruise phase are generally lower. Across the three different flight phases, the RBF Neural Network fuel consumption model consistently demonstrates the smallest prediction errors among the three models, indicating its superior prediction performance and stronger robustness.

In conclusion, the more input parameters the model has, the smaller the prediction errors, which further validates the robustness of the RBF Neural Network model. Comparing it to the set of key factors selected through various methods, the redefined sets of influencing factors based on the type of parameters and the ease of their acquisition exhibit deviations in the prediction performance after being input into the model. This highlights the importance of conducting an analysis of influencing factors.

### Ten-fold cross validation analysis

**Fig. 11:**
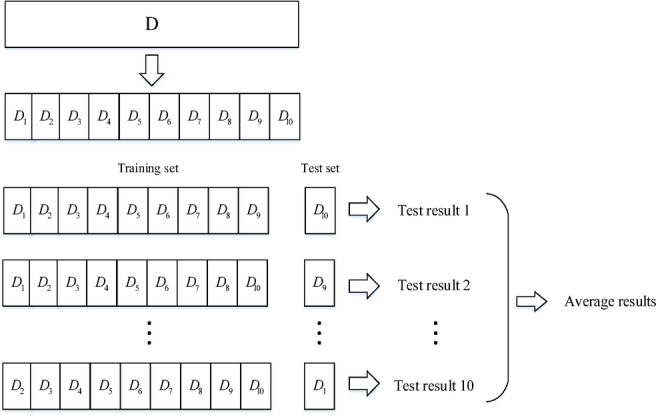
Schematic diagram of ten-fold cross verification.

In order to verify the robustness of the RBF Neural Network-based fuel consumption prediction model, a cross validation^26^ approach was employed. Cross validation is a statistical technique that involves dividing the data sample into smaller subsets. In this study, the ten-fold cross validation method was chosen. The total dataset was divided into 10 groups, and in each iteration, one subset was randomly selected as the test set, while the remaining nine subsets were combined to form the training set, as shown in Fig. 11.

In this study, a total of 44 flights are used in the QAR dataset. Therefore, for the ten-fold cross validation, the training set consisted of 40 flights, and the test set consisted of 4 flights. The results of the cross validation are presented in Table 8.

**Table 8:** Ten-fold cross validation results.

Serial number	Error rate during takeoff/climb phase	Error rate during cruise phase	Error rate during descent/approach phase
1	6.49%	3.60%	13.69%
2	5.72%	3.50%	13.91%
3	6.28%	3.67%	14.41%
4	6.13%	3.71%	14.12%
5	6.42%	3.84%	14.23%
6	6.04%	3.79%	14.18%
7	6.05%	3.78%	14.19%
8	5.47%	3.66%	13.44%
9	5.98%	3.84%	14.24%
10	6.19%	3.39%	13.89%
Average value	6.08%	3.68%	14.03%
Variance	0.000852%	0.000198%	0.000782%

From Table 8, it can be observed that the average error rate of the RBF Neural Network-based fuel consumption prediction model during the takeoff/climb phase, as determined through cross validation, is 6.08%. For the cruise phase, the average error rate is 3.68%, and for the descent/approach phase, the average error rate is 14.03%. The variances for all three phases are relatively small, further confirming the robustness of the model. In addition, the cross-validation results show minimal fluctuation in errors across different folds, indicating that the model does not rely on certain specific composition of the training or testing sets. This suggests greater reliability of the model in practical applications.

## Fuel consumption due to extra fuel

By utilizing the established fuel consumption prediction model and systematically varying the input parameters, different predictions of aircraft fuel consumption can be generated. This allows for the exploration of the relationship between carrying excess fuel and consuming excess fuel, while also enabling the evaluation of fuel efficiency.

### Analysis of aircraft fuel consumption phenomenon

As it is widely known, when other conditions are constant, a heavier aircraft will consume more fuel. Therefore, if more fuel is carried, the total weight of the aircraft is increased and, in turn, fuel consumption is increased. This phenomenon is known as the “fuel penalty for carrying additional fuel” phenomenon. By analyzing this phenomenon, we can discover the patterns of the “fuel penalty for carrying additional fuel” in aircraft.

In the composition of aircraft fuel, only the extra fuel is significantly influenced by human factors and meteorological conditions. The weight of extra fuel is primarily determined subjectively by the pilot or dispatcher based on daily weather conditions and other factors. Therefore, in this section, the “fuel penalty for carrying additional fuel” pattern mainly refers to the changes in aircraft fuel consumption resulting from variations in extra fuel. The objective is to study the relationship between these two variables and derive a formula to represent the “fuel penalty for carrying additional fuel” pattern, as shown in Eq. (3):

3 $$\:\varDelta\:y=f(\varDelta\:x)$$

where $\:\varDelta\:y$ represents the change in fuel consumption, $\:\varDelta\:x$ represents the change in extra fuel.

**Fig. 12:**
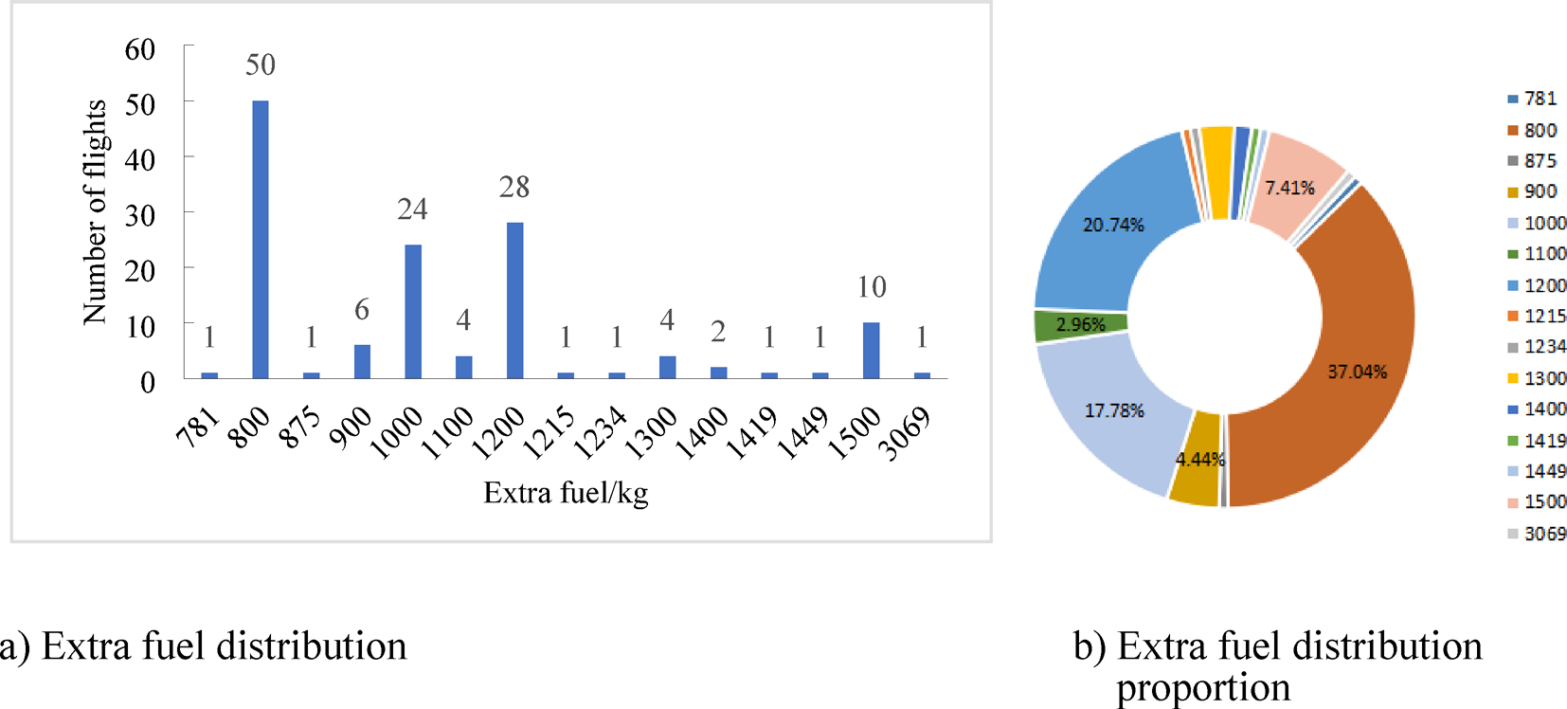
Statistics of extra fuel.

In order to analyze the relationship between extra fuel and fuel consumption, and to discover the “fuel penalty for carrying additional fuel” phenomenon in aircraft, we first conduct a preliminary analysis of the distribution of extra fuel. Based on the flight plan data of the 135 flights, we have compiled the main distribution of extra fuel, as shown in Fig. 12.

According to Fig. 12, among the 135 flights analyzed, the majority of flights (37.04%) carried an extra fuel load of 800 kg, followed by flights carrying 1200 kg (20.74%). Approximately 83.70% of the flights had an extra fuel load ranging from 800 kg to 1200 kg.

Subsequently, by systematically varying the weight of the extra fuel, the fuel consumption prediction model was employed to obtain the predicted results. Partial results are presented in Table 9.

In Table 9, the value of 0 represents no change in the extra fuel load. The corresponding fuel consumption prediction result at this point as the baseline. When the extra fuel load is modified, the difference between the modified fuel consumption prediction result and the baseline is calculated as the change in fuel consumption prediction result.

**Table 9:** Partial forecast results.

Extra fuel variation value	…	-10 kg		0	10 kg		…
Flight	…	Predicted values	The amount of change	Predicted values	Predicted values	The amount of change	…
1	…	6825.077	-0.796	6825.873	6826.669	0.796	…
2		6746.977	-0.796	6747.773	6748.568	0.796	
3		7020.093	-0.795	7020.888	7021.684	0.796	
4		6801.350	-0.797	6802.147	6802.942	0.796	
5		7192.984	-0.796	7193.780	7194.576	0.796	

**Fig. 13:**
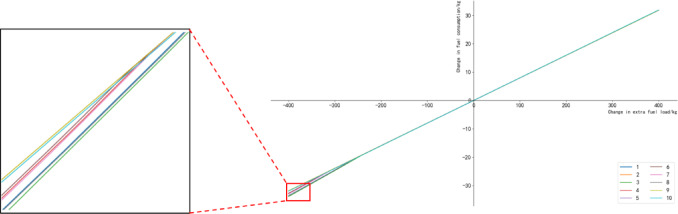
Changes of fuel penalty for carrying additional fuel load of some flights.

Then, the change in fuel consumption for selected flights is plotted in Fig. 13. The horizontal axis represents the change in extra fuel load, while the vertical axis represents the change in fuel consumption. It can be observed that the majority of flights exhibit a linear relationship, where the change in fuel consumption is proportional to the change in extra fuel load. By performing linear regression, the coefficient is determined to be c = 0.0809, and the goodness of fit is 0.99.

In summary, this section explored the relationship between the extra fuel load and aircraft fuel consumption by varying the amount of extra fuel carried by flights and utilizing the fuel consumption prediction model. Through linear regression analysis, the “fuel penalty for carrying additional fuel” phenomenon was characterized for the A321 aircraft type. The results showed that for every 100 kg increase in extra fuel load, the aircraft would consume an additional 8.09 kg of fuel, while a 100 kg decrease would result in a reduction of 8.09 kg of fuel consumption. This finding can contribute to more accurate calculation of fuel load during the pre-tactical phase, enabling precise control of fuel quantity for flights, reducing fuel consumption, minimizing carbon emissions, and ultimately saving fuel costs for airlines.

### Aircraft fuel efficiency evaluation

#### Fuel efficiency evaluation index

#####  Instantaneous fuel efficiency

The instantaneous fuel efficiency is defined as the ratio of true airspeed to fuel flow at a given time t. It represents the ability of an aircraft to generate a certain true airspeed per unit of fuel burned. It can be calculated using Eq. (4):

4 $$\:{SF}_{it}=\frac{{TAS}_{it}}{{FF}_{it}}$$

where $\:{SF}_{it}$ represents the instantaneous fuel efficiency of flight i at time t, measured in km/kg, $\:{TAS}_{it}$ represents the true airspeed of flight i at time t, measured in km/h, $\:{FF}_{it}$t represents the fuel flow of flight i at time t, measured in kg/h.

A higher instantaneous fuel efficiency indicates that the aircraft can achieve a greater speed with the same fuel consumption, resulting in a higher overall fuel efficiency. It primarily characterizes the fuel efficiency of an aircraft from an instantaneous and velocity perspective.

##### Cumulative fuel efficiency

The cumulative fuel efficiency at time t for an aircraft represents the ability of the aircraft to fly a certain distance per unit of fuel consumed. It is calculated as the ratio between the accumulated flight distance and the accumulated fuel consumption up to time t, as shown in Eq. (5):

5 $$\:{LF}_{it}=\frac{{L}_{it}}{{F}_{it}}$$

where $\:{LF}_{it}$ represents the cumulative fuel efficiency of flight i at time t, measured in km/kg, $\:{L}_{it}$ represents the cumulative flight distance of flight i at time t, measured in km, $\:{F}_{it}$ represents the cumulative fuel consumption of flight i at time t, measured in kg.

A higher value of cumulative fuel efficiency indicates that the aircraft can cover a greater distance with the same amount of fuel consumed, thereby indicating a higher fuel efficiency. This metric primarily characterizes the aircraft’s fuel efficiency from the perspectives of historical accumulation and flight distance.

#### Example analysis

In this section, a specific flight from the aforementioned 135 flights is selected as an example to conduct an analysis of fuel efficiency evaluation during flight using its QAR data.

**Fig. 14:**
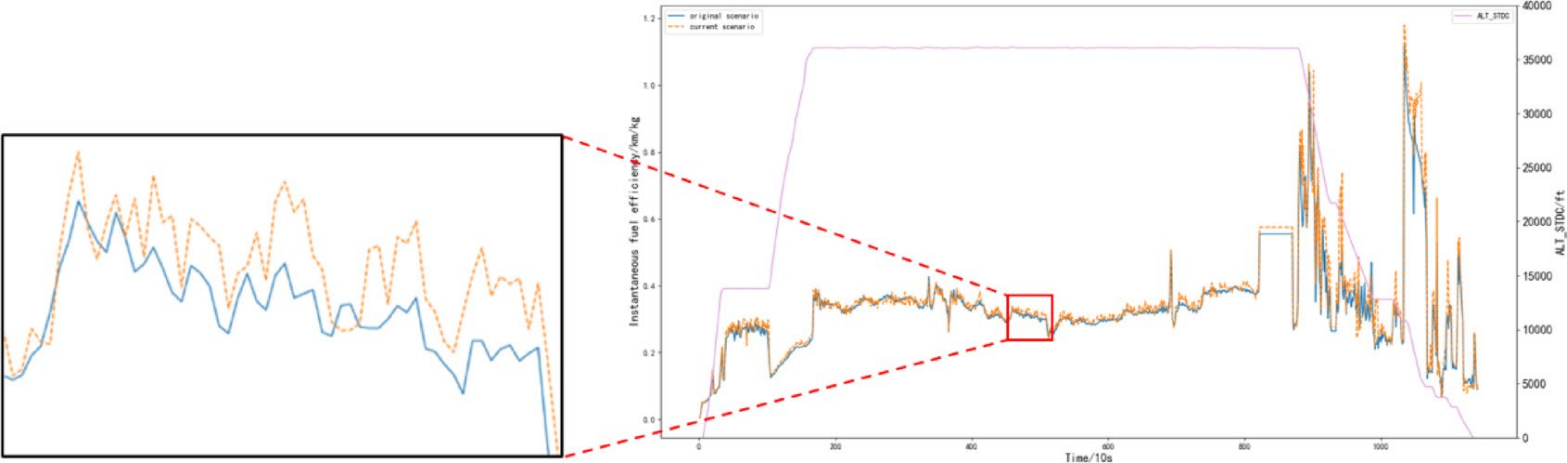
Instantaneous fuel efficiency of the flight.

**Fig. 15:**
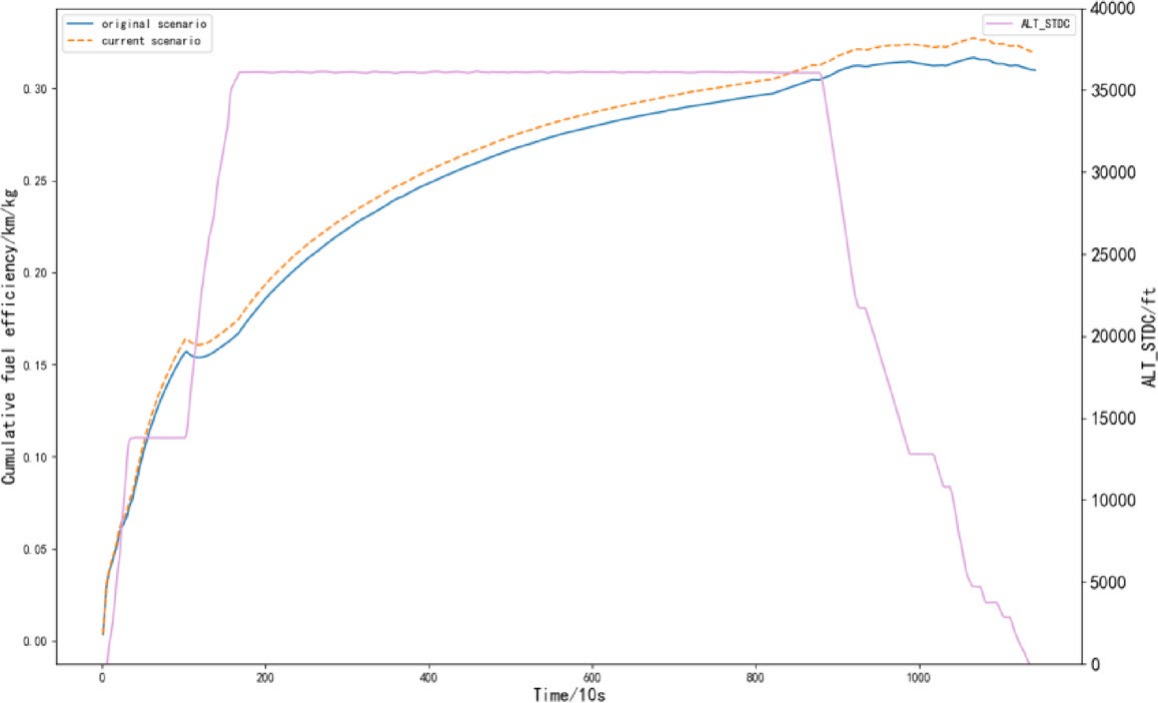
Cumulative fuel efficiency of the flight.

Since the evaluation of fuel efficiency during flight is conducted in real-time throughout the flight process, a phased fuel efficiency evaluation indicator is used. Based on the established fuel consumption prediction model, the QAR data of the selected flight is inputted, resulting in the predicted fuel consumption value (referred to as the “current scenario”). A comparison is then made between the predicted fuel consumption and the original QAR data (referred to as the “original scenario”) to evaluate the real-time instantaneous fuel efficiency and cumulative fuel efficiency of the flight. The results are presented in Figs. 14 and 15.

From Fig. 14, it can be observed that during the takeoff/climb phases as well as the cruise phase, the current scenario exhibits slightly higher instantaneous fuel efficiency compared to the original scenario, and the aircraft operates in a stable state overall. However, during the descent/approach phase, due to increased maneuvering and frequent changes in flight conditions, the fuel flow from the aircraft’s left and right engines becomes unstable, leading to fluctuations in the instantaneous fuel efficiency. Nevertheless, the current scenario remains largely consistent with the original scenario.

From Fig. 15, it can be observed that the overall cumulative fuel efficiency of the current scenario is slightly higher than that of the original scenario, and the aircraft operates in a stable state. This indicates that using the fuel consumption prediction model based on actual data can effectively reduce the aircraft’s fuel consumption and improve its fuel efficiency.

## Conclusion

This study developed an aircraft fuel consumption prediction model based on a lightweight and computationally efficient RBF Neural Network, achieving high-accuracy predictions across different flight phases. This study developed an aircraft fuel consumption prediction model based on a lightweight and computationally efficient RBF Neural Network, achieving high-accuracy predictions across different flight phases. The model effectively handles high-dimensional nonlinear data while maintaining low computational resource requirements, making it suitable for both pre-flight ground-based prediction and deployment in resource-constrained onboard environments. High-resolution, high-dimensional, and high-accuracy onboard QAR data were utilized to enhance the authenticity and completeness of the data, providing richer flight parameters compared to traditional sources. Considering the variability in external environmental factors and operational states across different flight phases, fuel consumption demonstrates distinct phase-dependent characteristics. To more accurately characterize the fuel consumption behavior inherent to each phase, a phase-segmented modeling approach is adopted, wherein dedicated predictive models are constructed for the takeoff/climb, cruise, and descent/approach flight segments. The model’s accuracy was validated through comparisons with CNN and MLP models, and its robustness was confirmed via ten-fold cross-validation. This provided an effective technical approach for aircraft fuel management with significant practical value. Furthermore, leveraging the prediction model, the study dynamically adjusted the amount of additional fuel carried and generated various fuel consumption prediction scenarios, thereby analyzing the “fuel penalty for carrying additional fuel” phenomenon. It also conducted pre-flight, in-flight, and post-flight fuel efficiency evaluations for specific flights. These efforts contributed to optimizing fuel loading strategies, reducing operational costs, and promoting energy conservation, emission reduction, and the development of green aviation.

Future research can further broaden the model’s applicability by extending it to different aircraft types and multiple flight routes, thereby enhancing its generalization capability and engineering adaptability. Additionally, the relationship of the “fuel penalty for carrying additional fuel” can be explored in greater depth under more complex and dynamic operational scenarios, with efforts to quantify its impact on flight performance and operational costs. Building on this, future work may also incorporate real-time flight planning information and operational constraints to further optimize fuel loading strategies. This would improve the intelligence of aircraft fuel management and promote the practical application of data-driven methods in flight operations and energy efficiency optimization.

## Data Availability

The data presented in this study are available on request from the corresponding author.
